# Hypoxic lung cancer cell-derived exosomal miR-21 mediates macrophage M2 polarization and promotes cancer cell proliferation through targeting IRF1

**DOI:** 10.1186/s12957-022-02706-y

**Published:** 2022-07-27

**Authors:** Jianxu Jin, Guiping Yu

**Affiliations:** 1 Department of Oncology, Xi’an traditional Chinese Medicine Hospital, Xi’an, 710021 Shanxi China; 2 Department of Oncology, Xi’an Ninth Hospital, Xi’an, 710054 Shanxi China

**Keywords:** Lung cancer, Hypoxia, Macrophage M2 polarization, Exosome, miR-21, IRF1

## Abstract

**Background:**

Hypoxia is the hallmark of the tumor microenvironment (TME) and plays a critical role during the progress of tumor development. A variety of microRNAs (miRNAs) transmitted by tumor-derived exosomes were involved in intercellular communication. We aimed to elucidate the precise mechanism by which tumor cell-derived exosomes promote lung cancer development by affecting macrophage polarization under hypoxic conditions.

**Methods:**

CD163 signal in tumor tissue from lung cancer patients was detected by immunohistochemical (IHC). The M2 polarization-related markers were assessed by flow cytometry and western blot. Exosomes were isolated from normoxic and hypoxic lung cancer cell culture and characterized by transmission electron microscope (TEM), dynamic light scattering (DLS), and western blot. RNA sequencing was performed to show the abnormally expressed miRNAs in exosomes from normoxic and hypoxic lung cancer cell culture. In addition, CCK-8 and clone formation assays were used to assess cell proliferation. Dual luciferase reporter assay was used to evaluate the relationship between miR-21 and IRF1. For in vivo experiment, the male nude mice were injected with H1299 cells with exosomes and miR-21 mimic treatment.

**Results:**

Firstly, we found a strong CD163 signal in tumor tissue from lung cancer patients by IHC. Subsequently, we co-cultured lung cancer cell line H1299 with M0 macrophage THP-1 and found that H1299 in a hypoxic environment promoted THP-1 M2 polarization. PKH67 fluorescence staining experiments confirmed that exosomes of H1299 origin were able to enter THP-1 and induced M2 polarization. RNA sequencing of exosomes showed that miR-21 level was significantly higher in the hypoxic culture group compared to the normoxic group. Subsequent cellular assays showed that miR-21 inhibited the expression of IRF1 by targeting it. In addition, the overexpression of IRF1 reversed the role of miR-21 on macrophage M2 polarization. Finally, we have confirmed through animal experiments that either hypoxic environment or high miR-21 level promoted tumor progression.

**Conclusions:**

High miR-21 level in hypoxic environments promoted macrophage M2 polarization and induced lung cancer progression through targeting IRF1.

## Background

Lung cancer is the most common cancer and the leading cause of morbidity and mortality among all malignancies worldwide [1–5]. Although advances in diagnostic and therapeutic techniques, including appropriate surgical resection, chemotherapy, and immunotherapy were used for clinical treatment, the 5-year survival rates were only 15% due to the presence of locally advanced or widely metastatic tumors majority of patients [6]. Therefore, the discovery of novel therapeutic targets and a better understanding of lung cancer molecular mechanisms are necessary.

Tumor microenvironment (TME) has been reported to play critical roles in tumor development, including immunosuppression, tumorigenesis, cancer growth, and metastasis [7]. Hypoxia, as a common phenomenon in the tumor microenvironment, could alter the tumor metabolism and subsequent effect on the development of cancer [8]. Hence, cells cultured under hypoxia condition can simulate the in vivo environment. More and more evidence has shown that hypoxia promotes cancer development through promoting cancer cell-secreted exosomes or vesicles [9–11]. Exosomes produced by many types of cells, including cancer cells, are one of the components of TME [12]. Exosomes derived from tumor cells containing variety of function mRNAs, miRNAs and proteins could transfer the genetic contents thus involved in intercellular communication [13]. Which exert a cancer promoting role through communicating with the tumor tissue and surrounding tissues and activating proliferation, metastasis and immune surveillance [14]. MiRNAs also have been reported expression dysregulation and as prognostic marker derived by tumor microenvironment [15].

Tumor-associated macrophage (TAM) is one of the immune cell populations of TME, which commonly leads to poor prognosis in patients with malignant tumors [16]. Macrophages are recognized to be divided into two main categories, classically activated macrophages (M1) and selectively activated macrophages (M2) [17]. M1 macrophages exhibit mainly pro-inflammatory activity, secreting a variety of pro-inflammatory factors such as tumor necrosis factor (TNF), nitric oxide, interleukin 1 (IL-1), and interleukin 12 (IL-12) [18]. M2 macrophages, on the other hand, exhibit a potent anti-inflammatory activity, antagonizing the pro-inflammatory response of M1 through upregulating IL-10 and downregulating IL-12. It also hydrolyses arginine to urea and ornithine by high expression of arginase-1 (Arg1), which acts as a precursor for proline and peptides, directly related to tissue damage repair and fibrosis. In addition, M2 macrophages can also highly express transforming growth factor-β (TGF-β), etc. And the specific markers of M2 include CD206, CD163, and CD68 [19]. Previous reports have been reported that macrophage M2 polarization derived by the tumor microenvironment, then polarized M2 macrophage promotes proliferation, invasion and EMT of tumor cells [20]. Researchers found that hypoxia induced tumor-associated macrophage enrichment and M2 polarization through HIFs. Downregulation of the expression of HIFs could inhibit the progression of glioma by reducing M2-polarized and TAM infiltration [21]. Moreover, pancreatic cancer cells generate exosomes delivery miR-301-3p in the hypoxic microenvironment, thus polarizing macrophages via activation of the PTEN/PI3Kγ signaling pathway. Polarized macrophages promote malignant behaviors of pancreatic cancer cells [22].

In this study, we found that hypoxic lung cancer cells promoted macrophage M2 polarization by secreting exosome transfer excessive miR-21. MiR-21 targeted binding the 3′UTR of interferon-regulatory factors 1 (IRF1) and downregulated the expression of IRF1 in macrophage. The polarized M2 macrophages further promoted lung cancer cell proliferation. In general, our research on the development mechanism of lung cancer might help to further provide new strategies for the treatment of lung cancer.

## Material and methods

### Clinical tissue samples

In this study, all clinical tissue samples, including eight pairs tumor samples and adjacent normal samples of NSCLC, were obtained from Xi’an traditional Chinese medicine hospital. Tissue samples are snap frozen in liquid nitrogen and stored at – 80 °C or fixed in 4% paraformaldehyde, as required for subsequent experiments. Every patient that participated in our research had been informed and signed consent before the study beginning. The study was approved by the Ethics Committee of Xi’an traditional Chinese medicine hospital and conducted according to the World Medical Association’s ethical principles.

### Immunohistochemical (IHC)

The IHC was performed according to previous method. Briefly, lung cancer tumor tissues were fixed and paraffin embedded, then cut into sections (4 μm). After multiple steps, including dewaxed and rehydrated, the sections were stained with mouse monoclonal antihuman CD163 antibody (1:1000, Abcam, USA) at 4 °C overnight, then incubated with the secondary antibody (1:2000, Abcam, USA) for 30 min at 37 °C, and finally counterstained with hematoxylin. An optical microscope (Olympus, Japan) was used for the sections observation.

### Cell culture and co-culture

Human non-small cell lung cancer cell line H1299 and human macrophage cell line THP-1 were purchased from Shanghai Academy of Sciences. H1299 and THP-1 cells were cultured with RPMI 1640 medium supplemented with 10% FBS, and 1% antibiotics, at 37 °C with 5% CO_2_. Prior to the start of the experiment, THP-1 monocytes are differentiated into macrophages by incubation with 100 nM phorbol 12-myristate 13-acetate (PMA, P8139, Sigma) for 48 h. The macrophages differentiated by PMA induction are named mTHP-1. When H1299 cells reached a density of 2 × 10^6^ cells/100 mm^2^, then cultured in the different O_2_ content environment, normoxic (20% O_2_) or hypoxic (1% O_2_). After cultured 24 h, the cells were used in subsequent experiments.

Transwell chambers (Corning, USA) containing 6.5-mm-diameter polycarbonate filter (1 μm pore) were used to co-culture H1299 cells and mTHP-1 cells. Hypoxia or normoxia H1299 cells were seeded in the upper compartments, mTHP-1 cells were in the under. When cells co-cultured 48 h, mTHP-1 cells were collected and identified the phenotype. Exosome inhibitor GW4869 (Sigma-Aldrich, USA) was diluted to 10 mM concentrations through cell cultured medium and used in the upper compartments to inhibit exosomes secretion. The mTHP-1 cells are cultured in a medium that containing with H1299 exosomes secreted under normoxia or hypoxia condition. The mTHP-1 cells were seeded in the upper compartments, H1299 cells in the lower. When cells were co-cultured for 48 h, H1299 cells were collected and detected cell proliferation.

### Flow cytometry to detect macrophage phenotype

THP-1 cells were incubated with PE anti-human CD163 and FITC anti-human CD206 antibody (BioLegend, USA) according to the manufacturers’ instructions. Briefly, we harvest, wash the cells and adjust cell concentration to 10^6^ cells/ml by using ice cold PBS solution supplemented with 10% FBS. Then in the dark, mTHP-1 cells were incubated with the appropriate conjugated primary antibody for 30 min at 4°C. Following cells were centrifuged at 400×*g* and resuspended by using ice cold PBS solution. Finally, analyze the cells using the Accuri C6 flow cytometer (BD Biosciences).

### Quantitative real-time PCR (RT-qPCR)

Total RNA was extracted from THP-1 macrophage, exosomes, and lung cancer tissues of animal model and then was reversed to cDNA by using PrimeScript RT reagent kit (Takara, Japan). RT-qPCR was performed with miRNA Universal SYBR qPCR Master Mix (Vazyme, China). U6 and GAPDH were used as internal reference genes. The RT-qPCR primers synthesized by TSINGKE were shown in Table 1.

**Table 1:** The sequence of RT-qPCR primers

Gene name	Forward primer (5′-3′)	Reverse primer (5′-3′)
TNF	ACTTTGGAGTGATCGGCCCC	TCGGGGTTCGAGAAGATGAT
Arg1	ACTTAAAGAACAAGAGTGTGATGTG	TCCAATTGCCAAACTGTGGTC
IL-10	GGCACCCAGTCTGAGAACAG	GGCAACCCAGGTAACCCTTA
TGF-β	GGAAATTGAGGGCTTTCGCC	CCGGTAGTGAACCCGTTGAT
GAPDH	ACGGATTTGGTCGTATTGGG	CGCTCCTGGAAGATGGTGAT
miR-21	CGGCGGTAGCTTATCAGACTGATGT	GTGCAGGGTCCGAGGT
miR-8485	CACACACACACACACACGUAU	ATACGTGTGTGTGTGTGTGTG
miR-3691-5p	AGUGGAUGAUGGAGACUCGGUAC	GTACCGAGTCTCCATCATCCACT
miR-4465	CUCAAGUAGUCUGACCAGGGGA	TCCCCTGGTCAGACTACTTGAG
miR-575	GAGCCAGUUGGACAGGAGC	GCTCCTGTCCAACTGGCTC
miR-320c	AAAAGCUGGGUUGAGAGGGU	ACCCTCTCAACCCAGCTTTT
miR-4761-5p	ACAAGGUGUGCAUGCCUGACC	GGTCAGGCATGCACACCTTGT
miR-19a-3p	AGUUUUGCAUAGUUGCACUACA	TGTAGTGCAACTATGCAAAACT
miR-19b-3p	UGUGCAAAUCCAUGCAAAACUGA	TCAGTTTTGCATGGATTTGCACA
miR-1285-3p	UCUGGGCAACAAAGUGAGACCU	AGGTCTCACTTTGTTGCCCAGA
U6	CTCGCTTCGGCAGCACA	AACGCTTCACGAATTTGCGT

### Exosome isolation and identification

When H1299 cells reached a density of 2 × 10^6^ cells/100 mm, cells medium was replaced by the containing 1% exosome-free serum (Life Technologies, USA) medium. The cells were cultured 24 h under hypoxia or normoxia conditions. Then, as previously described [23], the exosomes were isolated. In short, the cell culture supernatant was collected and centrifuged for several times at 4 °C, then after washing and resuspending, the exosomes were finally purified by sucrose-gradient centrifugation.

### Transmission electron microscope (TEM)

After isolation, the exosomes were diluted, fixed and then stained with phosphotungstic acid at room temperature, finally, observed with HT 7700 transmission electron microscope (Hitachi, Japan) at 80 kV.

Samples were stained with 2% phosphotungstic acid for 5 min at room temperature and air-dried, followed detected at 80 kV through the HT 7700 transmission electron microscope (Hitachi, Japan).

### Dynamic light scattering (DLS)

The 10-μl exosome was diluted and mixed well in 990 μl PBS buffer. The 1 ml exosome sample was measured and three independent readings were performed. High Performance Particle Sizer (Malvern, UK) was used in this experiment. For the data acquisition and analysis, Dispersion Technology Software configured for HPPS analysis was used.

### Western blot assays

Exosome marker protein was identified by western blot. The primary antibody including CD9 (1:1000, Abcam, USA), CD81 (1:2000, Abcam, USA), and TSG101 (1:1000, Abcam, USA). Goat anti-rabbit HRP conjugated antibody (1:2000, Abcam, USA) was used as a secondary antibody.

### Exosome labeling and tracking

The exosomes from H1299 cells that cultured in hypoxia condition were stained with PKH67 (Sigma-Aldrich, USA). THP-1 cells were inoculated into 24-well plates at the density of 5 × 10^5^ cells per well and incubated by culture medium containing PKH67-labeled exosomes (20 μg/ml). Four hours later, the cells were observed and photographed under the fluorescence microscope (Lecia, Germany).

### RNA sequencing

Firstly, preparing the small RNA sequencing library using NEBNext Multiplex Small RNA Library Prep Set for Illumina (NEB, USA). The Solexa CHASTITY quantity filtered reads were captured as Clean Reads by library sequencing. The RStudio software limma package was used for quantitative normalization and data processing. Using paired *t* test (*P* < 0.05) analyses the normalized intensity of each group (averaged normalized intensities of replicate samples, log2-transformed). Hierarchical clustering was done to display miRNAs with statistically significant differential expression between groups.

### Cell viability assay

The cell viability was determined by using Cell counting Kit-8 (CCK-8). Briefly, H1299 cells that co-cultured with different stimulated mTHP-1 cells were plated in 96-well plates (1 × 10^3^ cells/well) and cultured overnight. Then, 10 μL of CCK-8 reagent was added into H1299 cells and then cultured for 2 h at 37 °C. Absorbance at 450 nm of each sample was recorded in three independent experiments.

### Cell clone formation

The H1299 cells were co-cultured with THP-1 cells stimulated by normoxia and hypoxia H1299 exosomes or transfected miR-21 mimic, mimic NC, miR-21 inhibitor, inhibitor NC, separately. After co-culture, the H1299 cells digested with trypsin-collagenase, then seeded into 6-well plates and cultured with 5% CO_2_ at 37 °C. On the 14th day, H1299 cells were removed and washed, and then stained with crystal violet for 15 min. Finally, the clones number of the plate was counted.

### Western blot

The THP-1 cells that cultured with H1299 exosomes or transfected miR-21 mimics or inhibitor were collected. The protein was extracted with RIPA lysis buffer (Millipore, USA). Then BCA assay (Thermo Scientific, USA) was used for the quantity determining of total protein. Then, the protein dissolved in loading buffer was denatured and fractionated with 10% SDS-PAGE. Subsequently, the protein was transferred to PVDF membrane (millipore, USA), then incubated with 5% non-fat milk for 2 h, and finally incubated overnight with primary antibody at 4 °C, and following with secondary antibody for 2 h at room temperature. The primary antibody including IRF1 (1:1000, Abcam); PI3K (1:1000, Abcam); AKT (1:1000, CST); p-AKT (1:1000, CST); GAPDH, used as the internal control, (1:1000, Abcam). The secondary antibody is IgG (1:5000, Abcam). ImageJ software was used to quantify the protein bands.

### Cell transfection

The THP-1 cells were obtained and seeded (10^5^/ml) into 6-well plates and cultured overnight. Then THP-1 cells transfection was performed by using Lipofectamine 2000 (Thermo Scientific, USA) for miR-21 mimic, mimic control, miR-21 inhibitor, and inhibitor control transfection. THP-1 cells were collected in post-transfection 48 h for further analysis.

### Luciferase assays

The 3′UTR of IRF1, contained with the predicted binding sites and the mutated binding sites for miR-21, was amplification and inserted into luciferase reporter plasmid. The THP-1 cells were co-transfected IRF1 wild type luciferase plasmid or mutation luciferase plasmid and miR-21 mimic or inhibitor or corresponding control. The β-galactosidase plasmid was also transfected as an internal control. The luciferase signal was measured using luciferase assay kit (Thermo Scientific, USA).

### Animal model

The male nude mice obtained from the GemPharmatech Company for 6–8 weeks were used in this study. All animal experiments were approved by the Institutional Animal Care and Use Committees of Xi’an traditional Chinese medicine hospital and conformed to the NIH Guide. A total of 25 mice were randomly divided into 5 groups. Two of these groups were H1299 cells mixed with normoxic or hypoxic exosome-stimulated mTHP-1 cells (mTHP-1 + N and mTHP-1 + H). In addition, two other groups were H1299 mixed with normoxic exosome-stimulated and miR-21 mimic or NC mimic-infected mTHP-1 (mTHP-1-miR-21 mimic + N and mTHP-1-NC mimic + N). The mixed cells solution was injected into the mice subcutaneous of the right back flank. Tumor volume and weight were measured and recorded every 5 days. At last, the mice were euthanized after 20 days of injection, and the tumor tissues were obtained for later experiments.

### Statistical analysis

All the experiments in this study were repeated at least three times, and the obtained result data were expressed as mean ± standard deviation (SD). Student’s *t* test or one-way ANOVA was used to analyze the differences between the groups. All statistical analysis were performed by GraphPad Prism 7.0 or SPSS 13.0 statistical software. Additionally, *P* < 0.05 was considered significant statistically.

## Results

### Hypoxic lung cancer cells promote macrophage M2 polarization

**Fig. 1:**
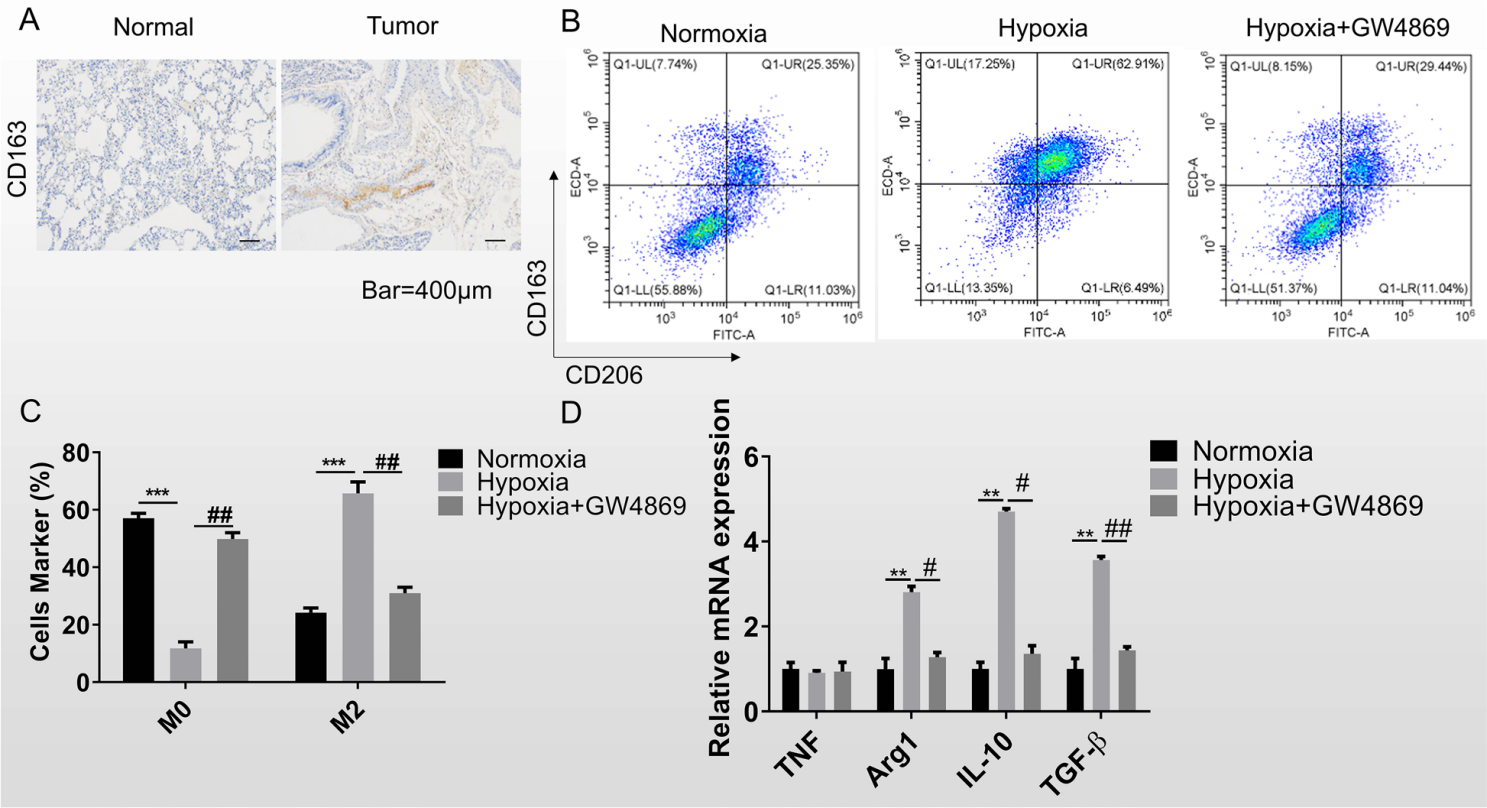
Hypoxic lung cancer cells promote macrophage M2 polarization. **A** IHC was performed to quantify the CD163 protein expression in clinical tissues. Scale bar = 400 μm. **B** Flow cytometry was used to detect the quantity of M2 macrophage through labeling the protein CD206 and CD163. GW4869 was used as the inhibitor of exosome secretion. **C** The quantitative analysis of flow cytometry results. **D** RT-qPCR was for the marker genes examining in mTHP-1 that co-cultured with the normoxia and hypoxia lung cancer cell H1299. Data was shown as mean ± SD. *n* = 3. *P < 0.05, **P < 0.01, ***P < 0.001

Macrophages, a kind of immune cells, their subpopulation M1 and M2 play different roles in cancer. To determine the macrophage phenotype, we used the IHC to detect the expression of M2 marker protein CD163 in lung cancer tissue and adjacent tissue. It was observed that CD163 positive cells were obviously more in tumor tissues than in adjacent tissues (Fig. 1A). This phenomenon suggested that the tumor microenvironment contributed to the M2 polarization of macrophages. To verify the effect of lung cancer cell on macrophage polarization, the cell culture of H1299 or hypoxic H1299 was used to add mTHP-1. Flow cytometry analysis showed that compared with the normoxic group, there was a notably more CD163^+^CD206^+^ cells in the hypoxic group. However, the percentage of CD163^+^CD206^+^ cells was apparently reduced after treatment with the exosome inhibitor GW4869 (Fig. 1B, C). This result suggested that hypoxic lung cancer cells secreted exosomes to induce M2 polarization in the tumor microenvironment.

Further, we examined the expression of several macrophage-related factors in mTHP-1 through RT-qPCR, including M1 macrophage marker (TNF), and M2 macrophage marker (Arg1, IL-10, TGF-β). The expression levels of Arg1, IL-10, and TGF-β were obviously increased in the hypoxic group, while decreased dramatically after treatment with GW4869 (Fig. 1D). These results suggested that hypoxic lung cancer cells promoted mTHP-1 toward M2 phenotype through secreting exosomes.

### Exosomes from hypoxic lung cancer cells promoted macrophage M2 polarization

**Fig. 2:**
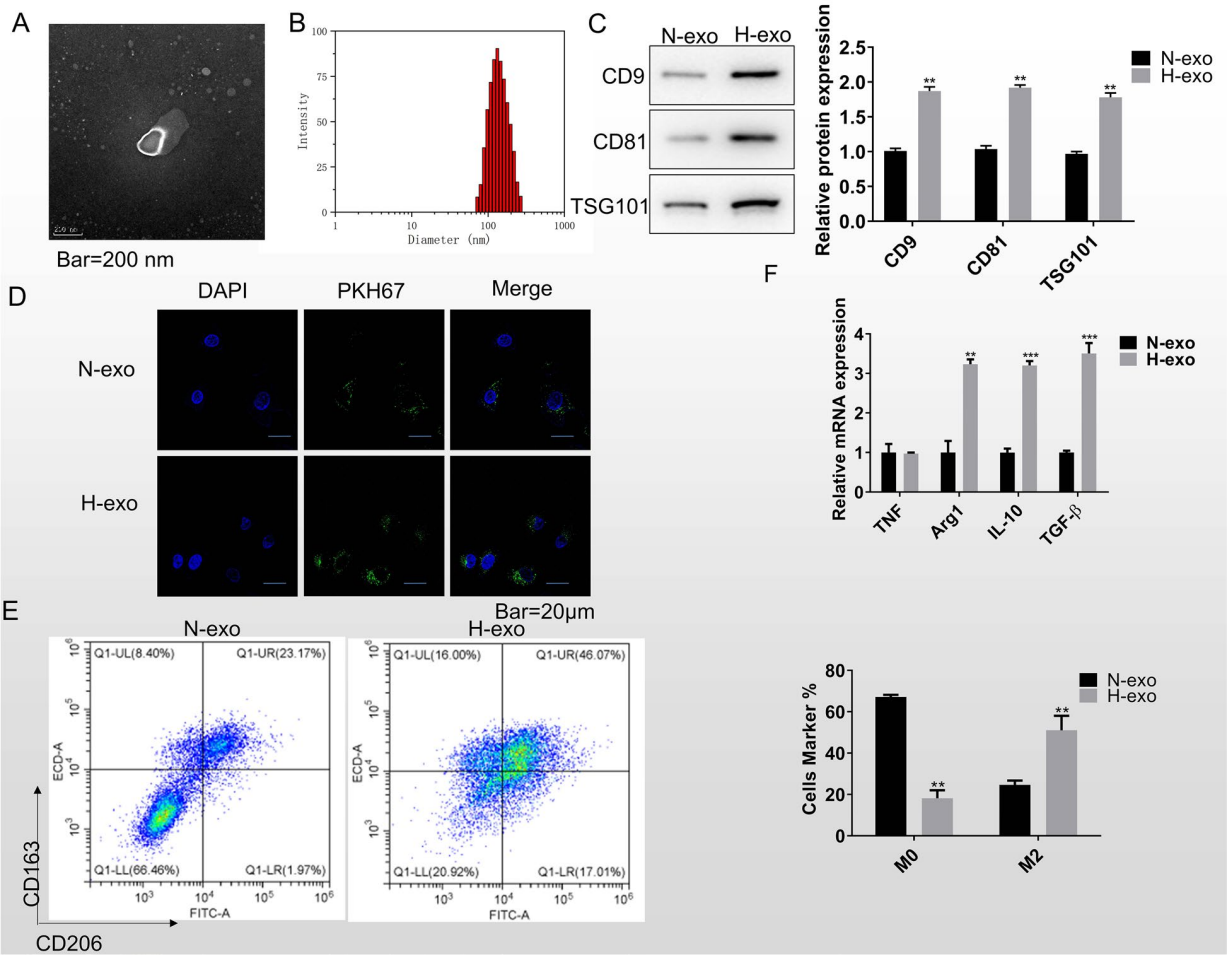
Exosomes from hypoxic lung cancer cells promote macrophage M2 polarization. **A** Exosome morphological characteristics were determined by transmission electron microscope. Scale bar = 200 nm. **B** Dynamic light scattering was used to detect the exosome size. **C** The expression of exosome protein biomarkers CD9, CD81, and TS101 was measured by western blot. **D** The immunofluorescence image shows the internalization of PKH67-labeled exosomes by macrophage. Scale bar = 20 μm. **E** Flow cytometry was used to detect the quantity of CD163^+^CD206^+^ M2 macrophage after treated with normoxia or hypoxia exosome for 48 h. **F** RT-qPCR was for the marker genes examining in macrophages that co-cultured with the normoxia and hypoxia exosomes, including TNF, Arg1, IL-10, and TGF-β. Data was expressed as mean ± SD. *n* = 3. *P < 0.05, **P < 0.01, ***P < 0.001

To further confirm the roles of exosomes in macrophage polarization, we collected exosomes from normoxic or hypoxic H1299 cells. The morphology and size of hypoxia exosomes were verified by TEM and DLS analysis (Fig. 2A, B). Positive expression of exosome markers CD9, CD81, TSG101 demonstrated the successful isolation of exosomes (Fig. 2C). Fluorescently labelled exosomes were co-cultured with mTHP-1. The results were shown in Fig. 2D, both exosomes from normoxic or hypoxic lung cancer cells could be absorbed by mTHP-1, suggesting that exosomes from lung cancer cells may have biological function on macrophage.

To confirm the function exosomes from lung cancer cells, we treated mTHP-1 with normoxic or hypoxic lung cancer cells-derived exosomes. The flow cytometry results showed that exosomes from hypoxic lung cancer cells promoted mTHP-1 M2 polarization (Fig. 2E). The RT-qPCR experiment also confirmed this conclusion (Fig. 2F). The above results confirmed that hypoxic lung cancer cells promoted macrophage M2 polarization by secreting exosomes.

### Exosomes promote macrophage M2 polarization through the PI3K/AKT signaling pathway and accelerate the proliferation of lung cancer cells

**Fig. 3:**
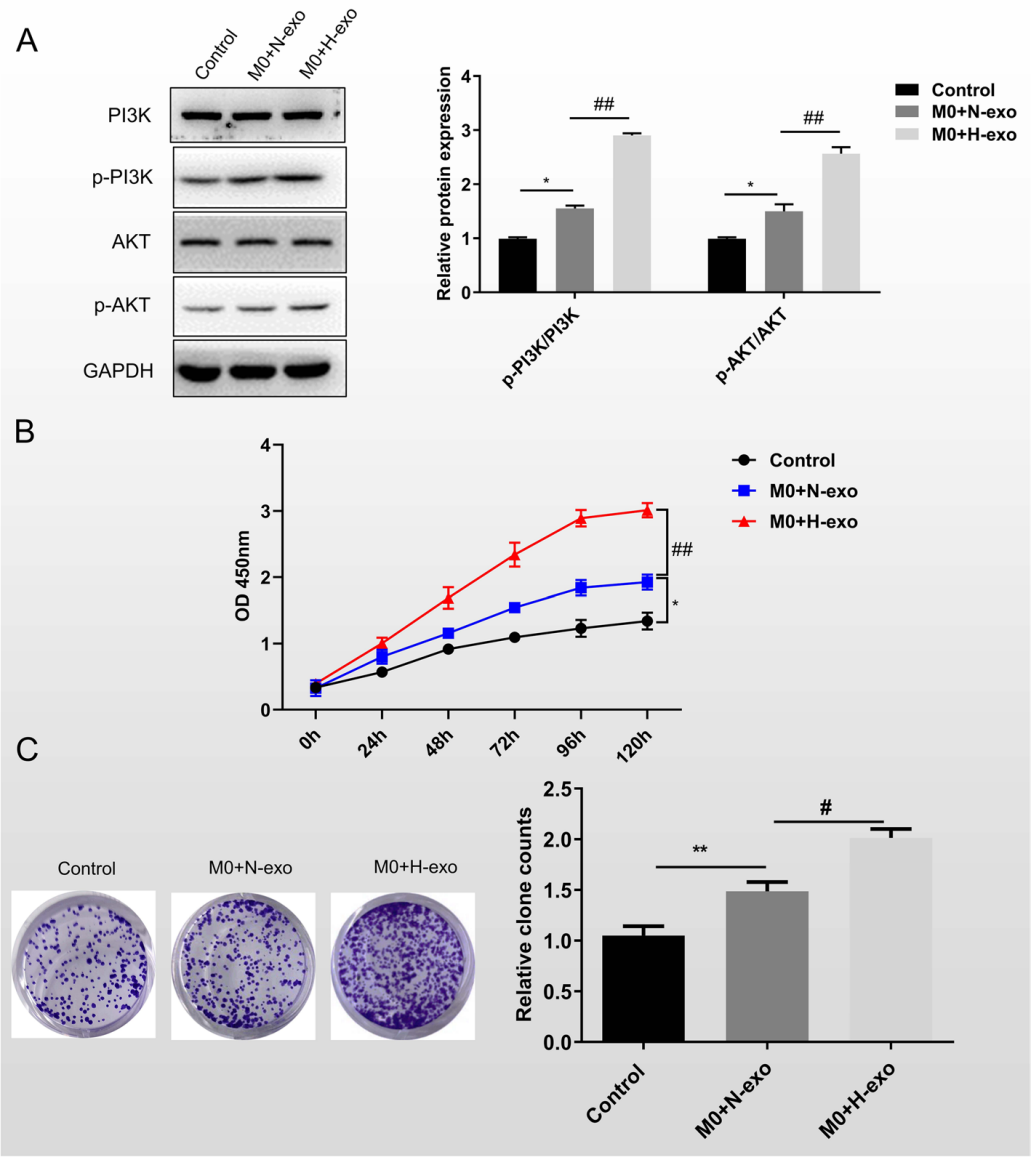
Exosomes promote macrophage M2 polarization through the PI3K/AKT signaling pathway and accelerate the proliferation of lung cancer cells. H1299 cells were cultured alone or co-cultured with the macrophage stimulated with PBS or exosome secreted from normoxia or hypoxia H1299 cells. **A**) Western blot was performed to detect the expression of PI3K/AKT signaling pathway associated genes. **B** H1299 proliferation level was analyzed through CCK8 assay and **C** clone formation assay. Data were expressed as mean ± SD. *n* = 3. *P < 0.05, **P < 0.01, ***P < 0.001

The PI3K/AKT pathway regulates multiple cellular functions, including macrophage polarization [24]. Therefore, we speculated that exosomes promoted macrophage polarization through PI3K/AKT signaling pathways. Western blot showed that p-PI3K and p-AKT expression upregulated in exosomes from hypoxic lung cancer cells compared with exosomes from normoxic lung cancer cells. The gene expression of normoxic exosomes co-cultured mTHP-1 with only little change compared with the group without exosomal stimulation (Fig. 3A). This result suggested that H1299-derived exosomes, especially those cultured in a hypoxic environment, promoted the activation of the PI3K/AKT signaling pathway.

To investigate the effect of lung cancer cell-derived exosomes on themselves through their involvement in macrophage M2 polarization in hypoxic environment, we co-cultured H1299 with mTHP-1 treated with exosomes derived from normoxic or hypoxic H1299. H1299 cultured with mTHP-1 induced by PBS was used as negative control. CCK8 assay showed that the proliferation rate of H1299 increased after co-culture with mTHP-1 after exosome stimulation (Fig. 3B). Moreover, the proliferation rate of H1299 in the hypoxic group was significantly higher than that in the normoxic group (Fig. 3B). Clone formation assay was used to verify this conclusion and the same trend of results was obtained (Fig. 3C). These data suggested that M2 macrophage induced by hypoxia H1299-derived exosomes feedback promoted the cell proliferation.

### Exosomal miR-21 promotes macrophage M2 polarization

**Fig. 4:**
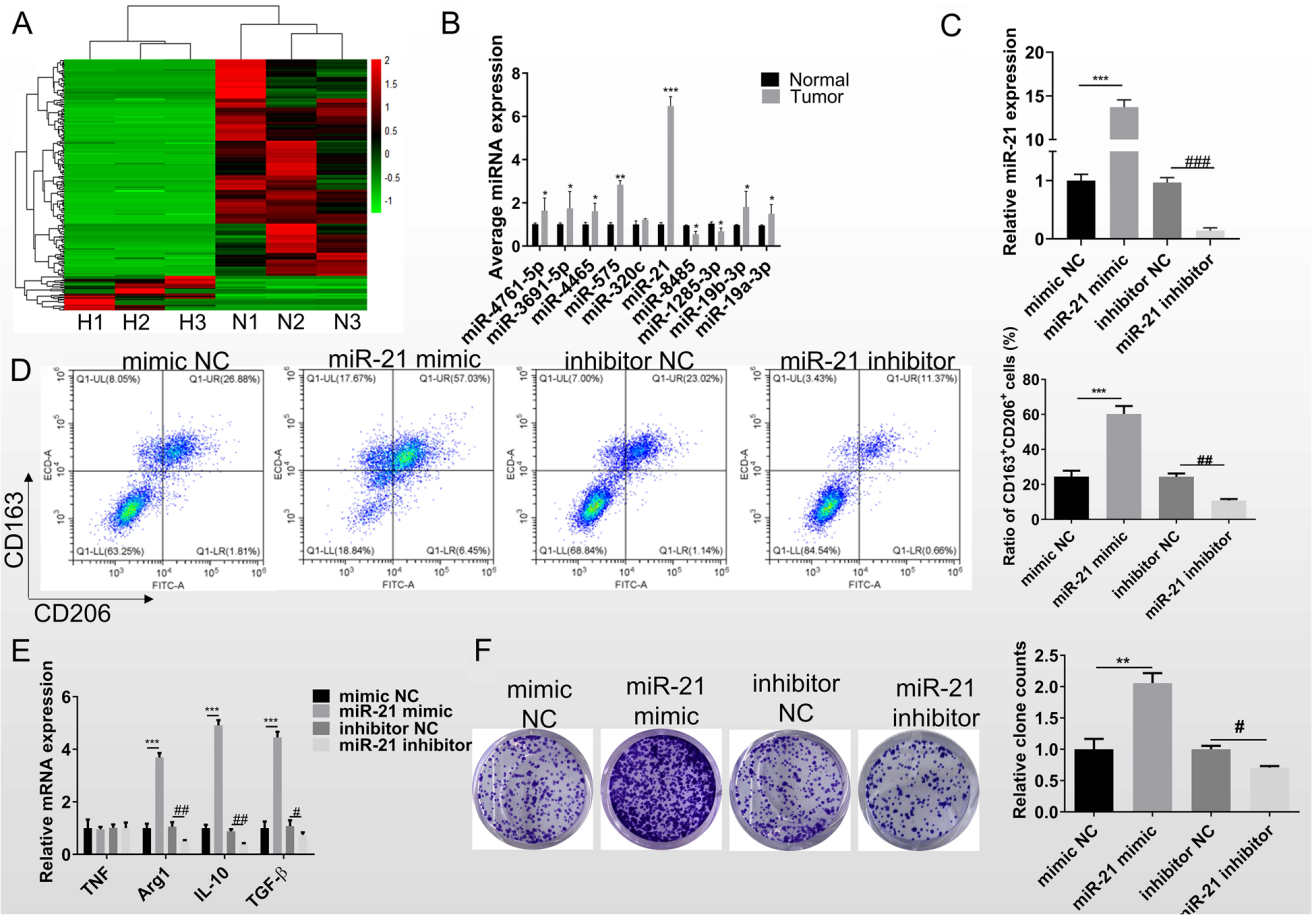
MiR-21 enriched in exosomes promotes macrophage M2 polarization. **A** RNA sequence was performed to analyze the different expression miRNAs between the exosomes secreted by H1299 cultured in normoxia (N) and hypoxia (H). **B** The top 10 differentially expressed miRNAs was validated by RT-qPCR in clinical tissues of lung cancer patients. The relative expressions were averaged and plotted. **C** The expression of miR-21 in mTHP-1 after transfected mimic or inhibitor and corresponding control was detected by RT-qPCR. **D** The quantity of CD163^+^CD206^+^ M2 macrophage after overexpress or knockdown miR-21 in mTHP-1 was by using flow cytometry. **E** RT-qPCR was for the marker genes examining in macrophages that co-cultured with the normoxia and hypoxia exosomes, including TNF, Arg1, IL-10, and TGF-β. **F** Clone formation assay of H1299 was assayed after co-culture with miR-21 knockdown or overexpression of mTHP-1. Data was expressed as mean ± SD. *n* = 3. *P < 0.05,**P < 0.01, ***P < 0.001

Exosome contains variety of RNA and protein. MiRNAs have been identified in exosomes and play roles via suppressing gene expression at the post-transcriptional levels [25]. To further reveal the underlying molecular of exosome on macrophage polarization, we performed RNA sequencing of exosomes from normoxic or hypoxic lung cancer cells, and the analysis results are shown in Fig. 4A. The top 10 differentially expressed miRNAs were validated by RT-qPCR in tumor and adjacent normal tissues. The results showed that miR-21 was the most significantly upregulated in cancer tissues (Fig. 4B). Therefore, in the later experiments we used miR-21 as the main object of study.

To further explore the function of miR-21 in macrophage polarization, we transfected miR-21 mimics, miR-21 inhibitor and corresponding contrast in mTHP-1, and following detected the transfection efficiency (Fig. 4C). Flow cytometry results showed that the proportion of CD163^+^CD206^+^ cells after miR-21 mimic was significantly increased, conversely, a significant decrease after miR-21 inhibitor treatment (Fig. 4D). The RT-qPCR results also displayed miR-21 increased M2 macrophage markers (Arg1, IL-10, and TGF-β) expression (Fig. 4E). Then H1299 cells were co-cultured with transfected mTHP-1. CCK-8 and clone formation assays showed that miR-21 overexpressed macrophage promoted lung cancer cell proliferation (Fig. 4F–G). The above results confirmed that miR-21 in exosomes secreted by lung cancer cells under hypoxic environment promoted macrophage M2 polarization, which in turn accelerated the proliferation of tumor cells.

### MiR-21 regulates M2 polarization by targeting IRF1

**Fig. 5:**
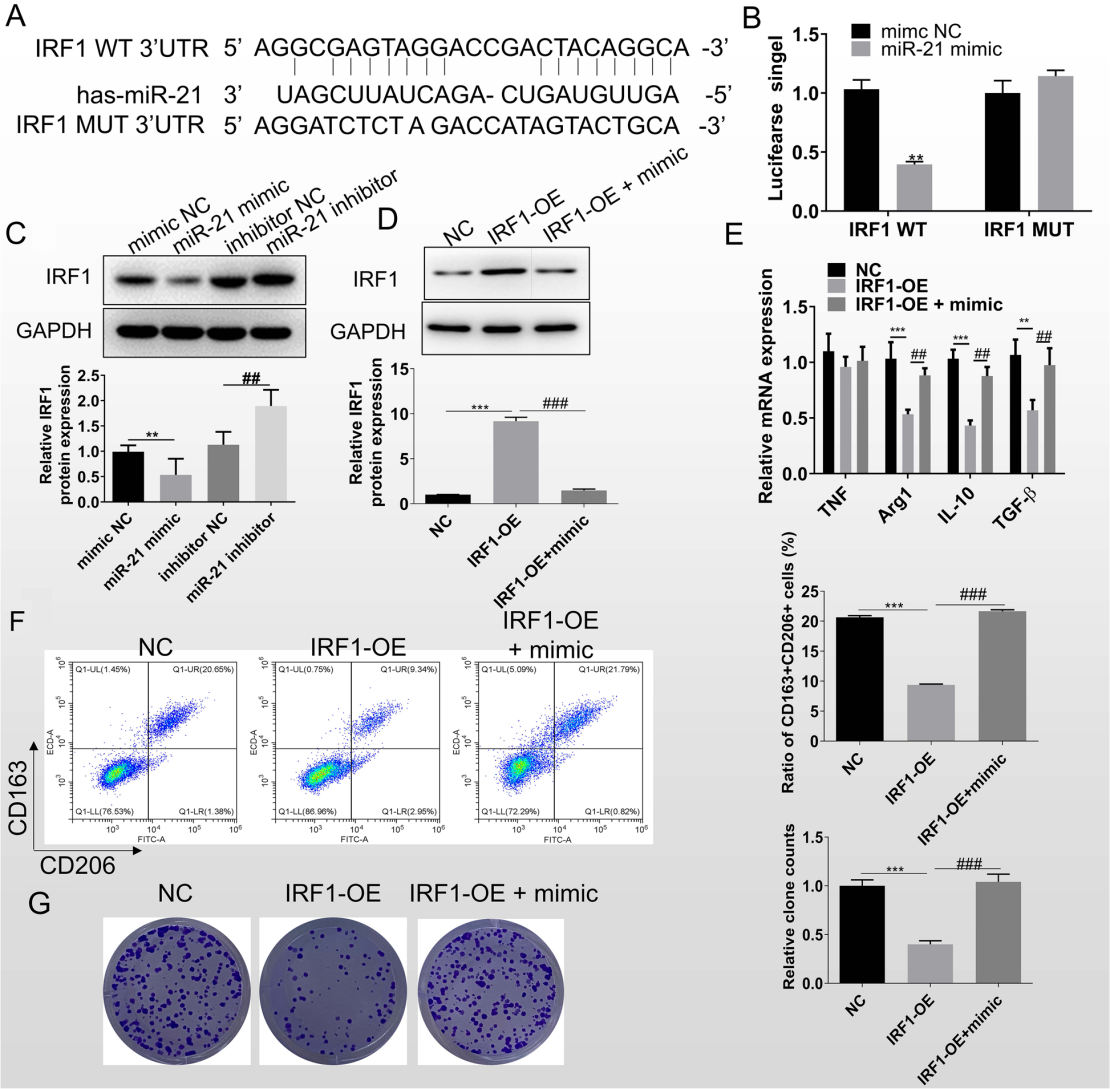
MiR-21 regulates M2 polarization by targeting IRF1. **A** The sequence of miR-21 binding sites in 3′UTR of IRF1. **B** The binding relationship between miR-21 and IRF1 was verified through luciferase reporter assay. **P < 0.01, compared with the mimic NC group. **C** Western blot was for IRF1 protein detecting. **P < 0.01, compared with the mimic NC group; ## *P* < 0.01, compared with the inhibitor NC group. **D** Western blot was for IRF1 protein detecting. **E** RT-qPCR detected the expression of TNF, Arg1, IL-10, and TGF-β in mTHP-1 after overexpression of IRF1 or both IRF1 and miR-21. **F** Flow cytometry was for the CD163^+^CD206^+^ M2 macrophage quantiting after overexpression of IRF1 or both IRF1 and miR-21. **G** Clone formation assay of H1299 was assayed after co-culture with mTHP-1 with overexpressed IRF1 or both IRF1 and miR-21. Data were expressed as mean ± SD. *n* = 3. *P < 0.05,**P < 0.01, ***P < 0.001

We identified IRF1 as a target gene for miR-21 by RNAhybrid tool site prediction (Fig. 5A). To verify whether IRF1 is a target of miR-21, mTHP-1 cells were transfected with a luciferase vector, IRF1 wild or mutant, and simultaneously treated with miR-21 mimic or inhibitor. Luciferase activity was significantly reduced or increased in IRF1 wild-type 3′UTR cells transfected with miR-21 mimic or miR-21 inhibitor, respectively, whereas no significant changes were found in the mutant 3′UTR group (Fig. 5B). The effect of miR-21 on IRF1 expression was examined by western blot, and the results showed that miR-21 negatively regulated the expression of IRF1 (Fig. 5C). These results confirmed that miR-21 targeted and negatively regulated the expression of IRF1.

We then explored whether IRF1 was involved in the process of miR-21 affecting mTHP-1 M2 polarization by assaying macrophage polarization-related indicators after altering IRF1 expression. We overexpressed IRF1 in mTHP-1 cells or overexpressed IRF1 while transfected with miR-21 mimic. The results of western blot showed that the expression of IRF1 was nearly tenfold in IRF1 overexpression group, while it was apparently reduced again after overexpression of miR-21 (Fig. 5D). Moreover, the expression levels of macrophage polarization-specific markers were examined by RT-qPCR, and we found that the expression levels of M2 polarization-related factors (Arg1, IL-10, and TGF-β) were significantly downregulated after overexpression of IRF1, while significantly upregulated after high expression of miR-21 (Fig. 5E). Similar results were obtained by flow cytometry, with a significant increase in the proportion of CD163^+^CD206^+^ cells after high expression of IRF1, which was counteracted by high expression of miR-21 (Fig. 5F). The above various results confirm the involvement of IRF1 as a target of miR-21 in the M2 polarization process. In addition, clone formation assay on H1299 after co-culture of these macrophages with H1299 showed that the clonogenic ability of H1299 was significantly reduced in the IRF1 high expression group, but was restored after high expression of miR-21 (Fig. 5G).

### Exosomal miR-21 facilitates cancer cell proliferation and induces macrophage M2 polarization in vivo

**Fig. 6:**
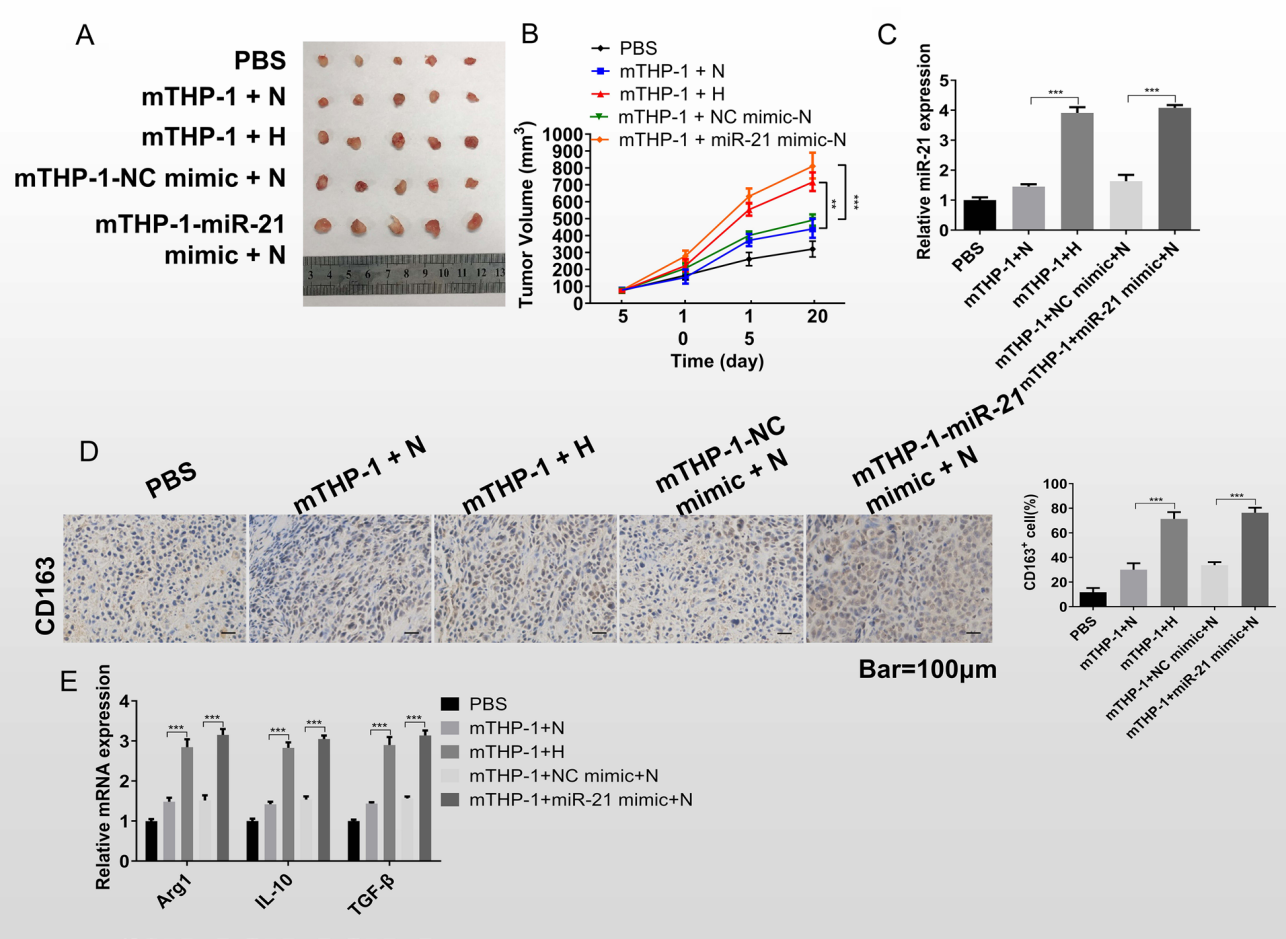
Exosomal miR-21 facilitates cancer cell proliferation and induces macrophage M2 polarization in vivo. H1299 cells mixed with or without the conditioned mTHP-1 stimulated by normoxia or hypoxia H1299 exosomes or transfected miR-21 mimic or NC mimic respectively. The mixed cells solution was injected into mice subcutaneous. **A** Photographs of tumors. **B** Growth curve of tumors. **C** The level of miR-21 in lung cancer tissues was detected by RT-qPCR assay. **D** IHC was performed to detect the CD163 expression in tumor tissues. Scale bar = 100 μm. **E** RT-qPCR assay was used to assess M2 polarization-related factors (Arg1, IL-10, and TGF-β) expression.*P < 0.05, **P < 0.01, ***P < 0.001

We validated the effect of hypoxic environment and miR-21 on macrophage M2 polarization and tumor development at the in vivo level by constructing mouse model of subcutaneous tumor. The results of animal experiments showed that after exosome stimulation from the hypoxic group, mTHP-1 accelerated the proliferation rate of H1299, and the tumor volume was markedly larger than that of the normoxia group. And miR-21 upregulated mTHP-1 promoted tumor growth rate even when stimulated by exosomes derived from hypoxic environments (Fig. 6A, B). RT-qPCR assay suggested that compared to the mTHP-1 + N group, the mTHP-1 + H group increased miR-21 expression; while compared to the mTHP-1-NC mimic + N group, the mTHP-1-miR-21 mimic + N group also upregulated miR-21 level in lung cancer tissues (Fig. 6C).

Subsequently, IHC results of the tumor tissues showed that compared with the mTHP-1 + N group, the CD163 positive cells in the tumor tissues of the mTHP-1 + H group were prominently more. And in the normoxia-derived exosome stimulation, the presence of CD163 positive cells was significantly higher in the miR-21 high expression group than in the control group (Fig. 6D). Furthermore, RT-qPCR assay suggested that compared to the mTHP-1 + N group, the mTHP-1 + H group increased M2 polarization-related factors (Arg1, IL-10, and TGF-β) expression; while compared to the mTHP-1-NC mimic + N group, the mTHP-1-miR-21 mimic + N group also upregulated M2 polarization-related factors (Arg1, IL-10, and TGF-β) levels (Fig. 6E). The results of animal experiments demonstrated that miR-21 content in exosomes secreted by lung cancer cells under hypoxic environment was high, which could accelerate tumor progression by promoting macrophage M2 polarization.

## Discussion

TME has received more attention to its function in tumor development and procession. Previous studies have reported that TME containing malignant tumor cells, macrophage, cancer-associated fibroblasts and other variety kind cells. Each of these cell types has capabilities that effect TME through secreted cell cytokines or other substances [26]. Hypoxia, as one of the most critical features of TME, is related to tumor growth, tumor metastasis, tumor angiogenesis, and poor prognosis of patients [27]. So, exploring the roles of hypoxia in tumor procession and finding a treatment that target hypoxia-induced pathways is necessary.

Exosomes are secreted by cells and have been reported to play crucial roles in the interaction of cells and TME. For example, tumor cells deliver abundant proteins and nucleic acids to mesenchymal through secreting exosomes. Exosome was uptake by MSCs and changed the cell function, then promote tumor development [28]. Chen et al. found exosomes derived from epithelial ovarian cancer cells remodel macrophages to tumor-associated macrophages, and hypoxic microenvironments could facilitate this process. Tumor-associated macrophages promote tumor proliferation and migration in a feedback loop [29]. M2 phenotype macrophages can promote tumor progression by secreting anti-inflammatory cytokines for tissue regeneration and immunosuppression. Because of this, tumor associated macrophages usually exhibit M2-like phenotype [30]. In head and neck cancer, EMT transcription factor Snail promotes macrophage M2 polarization by inducing tumor cells-secreted exosomes riched with miR-21 [31]. Herein, we found that exosomes from hypoxic lung cancer cells could promote macrophage M2 polarization Moreover, we further found polarized M2 macrophage feedback to lung cancer proliferation.

MiRNAs, as a class of endogenous, have multiple important regulatory roles within cells, which is mainly due to the fact that each miRNA can target binding with multiple downstream genes, and several miRNAs can also regulate a same gene [32]. It is hypothesized that miRNAs regulate one third of all human genes. First discovered in 1993, miRNA research has moved from the laboratory to the clinical stage in less than 30 years [33]. Some miRNA therapeutics in clinical trials include Mirvirasen (Santaris Pharma A/S and Hoffmann-La Roche), RG-101 (Regulus Therapeutics), MRG-106 (miRagen Therapeutics), MesomiR-1 (EnGeneIC), and others [34]. Several studies clarified that hypoxic cancer cell-derived exosomal miRNAs participated in the development of human cancer [35]. For instance, exosomal miR-23a from hypoxic lung cancer cells promoted angiogenesis and vascular permeability, thereby accelerating tumor lung cancer progression [35]. Besides, hypoxic lung cancer cell-secreted exosomal miR-582-3p promoted the development of lung cancer [36]. However, [37] whether hypoxic lung cancer cell-derived exosomal miRNAs played important roles on macrophage polarization has never been reported. Here, we found that exosomes from hypoxic lung cancer cells were enriched in miR-21.

Currently, miR-21 has been reported in several articles and is associated with disease progression in colon cancer, breast cancer, renal fibrosis, cardiac fibrosis by targeting PTEN, PDCD4, SMAD7 and SPRY, and others [38–41]. For instance, Dai L et al. found that miR-21 promoted growth and EMT in lung cancer cells via PTEN/Akt/GSK3β signaling [42]. Li H et al. also suggested that miR-21 accelerated lung cancer cell growth and metastasis [43]. Studies suggested that miR-21-5p regulated the macrophage M2 polarization and the macrophage-mediated pro-inflammatory response [37, 44, 45]. For instance, Xue J et al. clarified that miR-21 promoted macrophage M2 polarization in arsenicosis-induced hepatic fibrosis [37]. Caescu CI et al. reported that activation of CSF-1R on mouse macrophage induced M2 polarization and inflammatory responses by increasing miR-21-5p level [45]. Furthermore, exosomal miR-21-5p from hypoxia pre-challenged mesenchymal stem cells promoted lung cancer cell growth and mobility and macrophage M2 polarization [44]. By using mimic and inhibitor, we demonstrated that miR-21 promoted macrophage M2 polarization and accelerated tumor progression.

Several studies have shown that IRF1 is regulated by some miRNAs and plays a vital role in macrophage polarization [46–48]. For instance, Zhu X et al found that miR-19a-3p inhibited M1 macrophage polarization through STAT1/IRF1 pathway [46]. Shi Y et al. showed that miR-106b-5p promoted M2 macrophage polarization through suppressing IRF1/IFN-β signaling in glioblastoma [47]. Besides, miR-130b-3p promoted M2 macrophage polarization through directly targeting IRF1 [48]. Our study identified IRF1 as a downstream target of miR-21. Furthermore, the overexpression of IRF1 reversed the role of miR-21 on macrophage polarization.

## Conclusion

**Fig. 7:**
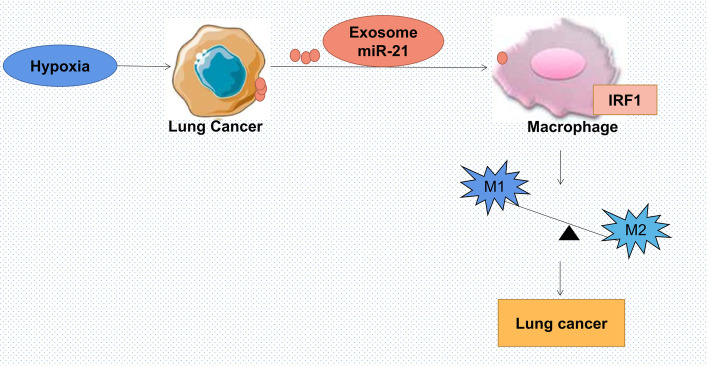
Proposed model for summarizing the main conclusions

To the best of our knowledge, the present study was the first to identify the important extracellular signaling molecules exosomal miR-21 involved in the associated mechanism between hypoxic lung cancer cells and macrophages. In conclusion, hypoxic lung cancer cell-derived exosomal miR-21 promoted macrophage M2 polarization and induced lung cancer progression through targeting IRF1 (Fig. 7). These findings shed light on the communication of hypoxic cancer cells and macrophages in the process of lung cancer development. And it may provide new targets and pathways for the therapy of lung cancer.

## Data Availability

The data used to support the findings of this study are available from the corresponding author upon request.

## Abbreviations

TEM: Tumor microenvironment
miRNAs: microRNAs
IRF1: Interferon regulatory factor 1
TAM: Tumor-associated macrophage
TNF: Tumor necrosis factor
IL-1: Interleukin 1
IL-12: Interleukin 12
Arg1: Arginase-1
TGF-β: Transforming growth factor-β
HIFs: Hypoxia-inducible factors
PTEN: Phosphatase and tensin homolog deleted on chromosome ten
PI3Kγ: Phosphoinositide 3-kinase gamma
NSCLC: Non-small cell lung cancer
IHC: Immunohistochemical
FBS: Fetal bovine serum
RT-qPCR: Quantitative real-time PCR
TEM: Transmission electron microscope
DLS: Dynamic light scattering
3′UTR: 3′ untranslated region
CCK8: Cell counting kit-8
PDCD4: Programmed cell death 4
SMAD7: SMAD Family Member 7
SPRY: SPla/Ryanodine receptor
